# Utility of Clinical Signs in the Diagnosis of Testicular Torsion in Pediatric Age: Optimization of Timing in a Time-Sensitive Pathology

**DOI:** 10.3390/children12091220

**Published:** 2025-09-12

**Authors:** Fabiola Cassaro, Salvatore Arena, Roberta Bonfiglio, Angela Alibrandi, Santi D’Antoni, Carmelo Romeo, Pietro Impellizzeri

**Affiliations:** 1 Pediatric Surgery Unit, Department of Human Pathology in Adulthood and Childhood “G. Barresi”, Messina 98125, Italy; fabiola.cassaro@studenti.unime.it (F.C.); roberta.bonfiglio@studenti.unime.it (R.B.); santi.dantoni@studenti.unime.it (S.D.); romeoc@unime.it (C.R.); pietro.impellizzeri@unime.it (P.I.); 2 Department of Biomedical and Dental Sciences and Morpho-Functional Imaging, University of Messina, Messina 98125, Italy; 3 Department of Human Pathology in Adulthood and Childhood “G. Barresi”, Messina 98125, Italy; angela.alibrandi@unime.it

**Keywords:** acute scrotum, testicular torsion, twist score, Doppler ultrasound, children, pediatric patients, clinical diagnosis

## Abstract

**Background/Objectives**: Acute scrotal pain in children and adolescents requires prompt evaluation to differentiate testicular torsion from other scrotal pathologies. Testicular torsion, a surgical emergency, can lead to irreversible testicular damage if not managed rapidly. This study aims to assess the clinical signs, diagnostic tools, and outcomes related to testicular torsion in patients presenting with acute scrotal pain. **Methods**: A retrospective analysis was conducted on 111 patients diagnosed with acute scrotal conditions. Clinical signs, presentation times, ultrasound findings, and treatment outcomes (surgical intervention, orchiectomy, or medical management) were evaluated. The statistical analysis was performed with a *p*-value < 0.05 being considered significant. Correlations between clinical signs, diagnostic imaging, and treatment modalities were assessed. **Results**: The most prevalent clinical signs were scrotal swelling (92.2%), pain on palpation (93.5%), and scrotal hyperemia (84.4%). Testicular torsion was strongly associated with the absence of the cremasteric reflex (*p* < 0.0001) and testicular retraction (*p* < 0.0001). Ultrasound findings, including absent blood flow and testicular heterogeneity, were highly predictive of surgical intervention (*p* < 0.01). Patients presenting within 8 h of symptom onset had higher success rates of detorsion and testicular preservation. **Conclusions**: Timely and accurate clinical assessment, including the identification of key signs such as the absence of the cremasteric reflex and testicular retraction, is critical for differentiating testicular torsion from other conditions. Ultrasound findings are pivotal in guiding treatment decisions in cases of clinical uncertainty. Early intervention significantly improves testicular viability and outcomes, underscoring the importance of rapid diagnosis and management.

## 1. Introduction

Acute scrotum is the most common urological emergency in the pediatric population, typically presenting with scrotal pain, erythema, and swelling [1]. A variety of underlying conditions may be responsible, including trauma, acute epididymitis, pyocele, vasculitis, tension hydrocele, incarcerated inguinal hernia, varicocele, torsion of a testicular appendage, and most critically, torsion of the spermatic cord [2]. Torsion of the spermatic cord is particularly concerning due to its diagnostic challenge and the significant risk of compromised testicular perfusion, which may result in ischemia and irreversible tissue damage [3]. Prompt identification and accurate differentiation of the underlying cause of acute scrotum are essential to ensure timely surgical intervention and preserve testicular viability. Reported testicular salvage rates in cases of torsion range from 30% to 70% [4]. Exact etiology of acute scrotum can be challenging, as clinical signs and physical examination findings often overlap. To aid in this diagnostic process, Barbosa et al. developed the TWIST score [5]. This scoring system demonstrates strong diagnostic accuracy and helps minimize the reliance on scrotal ultrasonography. In low-risk cases, testicular torsion can be safely excluded, whereas high-risk patients should undergo immediate surgical exploration. Those classified as intermediate risk may benefit from further evaluation with scrotal ultrasound [1].

However, institutional guidelines regarding scrotal imaging may vary. While scrotal Doppler ultrasound is a fairly accurate, highly sensitive, and specific tool for diagnosing testicular torsion, helping to avoid unnecessary surgeries, it can be unhelpful when testicular torsion is strongly suspected. In such time-sensitive situations, any delay caused by imaging could be critical, as prompt surgical intervention is essential for the best possible outcomes [6,7,8].

This retrospective study aimed to pinpoint the clinical signs and symptoms most indicative of an acute scrotum diagnosis and to assess their relationship with treatment outcomes, ultimately to support quicker and more appropriate therapeutic choices.

## 2. Materials and Methods

The study was conducted in accordance with the Declaration of Helsinki, and the protocol was approved by the Ethics Committee of A.O.U. “G.Martino” (IRB approval no. 82–21, 1 December 2021). This retrospective cohort study included pediatric patients who were admitted to our institution with a clinical diagnosis of “acute scrotum” over an eight-year period.

### 2.1. Diagnostic Evaluation and Management

Acute scrotum is defined as new onset testicular pain, scrotal swelling, and/or tenderness of intrascrotal contents, and diagnosis is made based on the patient’s clinical history and a physical examination. A Doppler ultrasound examination was performed in all suspected cases of acute scrotum, as radiology coverage is guaranteed 24/7. Key clinical parameters taken into account included the onset of pain, tenderness on palpation, presence of scrotal swelling and erythema (hyperemia), absence of the cremasteric reflex, firm testis, and high riding testis. If spermatic cord torsion was suspected, surgical intervention was prioritized. Otherwise, medical management was tailored based on the clinical and/or ultrasound findings.

Initial evaluation was performed either by pediatric emergency physicians, with immediate pediatric surgical consultation when testicular torsion was suspected.

The follow-up schedule included clinical evaluations at 1 week, 1 month, 6 months, and 1 year postoperatively and subsequently annually until transition to adulthood or as needed, based on the referring physician’s recommendation or upon patient request. Ultrasound assessments were performed at 1 month, 6 months, and 1 year. In cases of orchiectomy, the placement of a testicular prosthesis was offered to the patient and/or their parents, and the timing of the procedure was discussed with them.

### 2.2. Data Collection

Data was gathered retrospectively from electronic medical records, including surgical notes and radiological reports. The information collected included patient demographics, details about their clinical presentation, and characteristics from the Doppler ultrasound examination. The latter assessment encompassed evaluation of testicular enlargement, the homogeneity of the parenchymal tissue, the integrity of vascular signals, and the presence of the “whirlpool sign”. Patients with missing data for a given variable were excluded from the analysis of that specific parameter but retained for inclusion in other statistical evaluations.

### 2.3. Outcomes

The main goal was to see how well the clinical signs and Doppler ultrasound evaluations matched the final diagnosis and chosen treatment.

### 2.4. Statistical Analysis

Statistical analysis was conducted using IBM SPSS Statistics version 25 [9]. Pearson’s chi-square test was employed, with statistical significance set at *p* < 0.05. Patients with gaps in data for certain variables were excluded from those specific analyses but remained in the study for other statistical assessments. Correlations between clinical signs and treatment outcomes were calculated to determine how accurate individual symptoms were in diagnosing conditions and predicting whether surgical or medical management would be needed.

## 3. Results

One hundred eleven patients were enrolled in the study, with ages ranging from 2 to 19 years (mean age: 10.7 years ± 4.3). A 19-year-old male was admitted with the diagnosis of acute scrotum at our institution, and was admitted to our pediatric surgery department as he had previously been followed for recurrent testicular pain. Common clinical presentations included scrotal swelling in 95 out of 103 patients (92.2%; 8 missing data points), tenderness on palpation reported by 100 out of 107 patients (93.5%; 4 missing data points), firm testis observed in 43 out of 65 patients (66.2%; 46 missing data points), scrotal hyperemia noted in 65 out of 77 patients (84.4%; 34 missing data points), absence of the cremasteric reflex found in 25 out of 38 patients (65.8%; 73 missing data points), and high riding testis seen in 13 out of 41 patients (31.7%; 70 missing data points). These findings are detailed in Table 1.

Testicular torsion was diagnosed intraoperatively in 44 out of 110 patients (39.6%, with one missing data point).

Patient age proved to be a significant diagnostic factor. Specifically, patients older than 10.77 years had a higher likelihood of experiencing testicular torsion (*p* < 0.001), whereas younger patients were more predisposed to non-torsion conditions (*p* < 0.001). An analysis of presentation timing showed that 40 out of 101 patients (39.6%, with 10 missing data points) were admitted at the emergency department within 8 h of symptom onset. Conversely, 61 out of 101 patients (60.4%, with 10 missing data points) presented after 8 h.

Diagnostic correlations indicated that spermatic cord torsion was strongly associated with both the absence of the cremasteric reflex (*p* < 0.0001) and high riding testis (*p* < 0.0001); this also reflects their negative correlation with non-surgical management. Conversely, both of these signs were negatively correlated with non-torsion diagnoses (*p* < 0.0001). Patients who were admitted after 8 h after symptom onset exhibited a higher frequency of orchiectomy (*p* = 0.001). Orchiectomy was also strongly correlated with the absence of the cremasteric reflex (*p* < 0.0001) and firm testis (*p* = 0.003), suggesting that these signs point to testicular torsion needing surgery. No significant correlation was found for other studied clinical signs. Treatment analysis revealed significant associations with the presence of these key features, while no significant correlations were observed for the remaining signs (Table 2).

Surgical intervention was performed in 61 out of 111 patients (55.0%). Testicular torsion was confirmed in 44 cases, of which 19 out of 111 (17.1%) required orchiectomy and 25 underwent successful detorsion with gonadal preservation. In the remaining 17 surgically explored patients, no spermatic cord torsion was found. The other diagnoses were: 5 orchidymitis cases, 8 cases of torsion of the testicular appendage, and 4 post-traumatic hematoma cases.

The decision to proceed with orchidectomy was made intraoperatively in cases where the testis appeared macroscopically necrotic and the intraoperative bleeding test was negative.

Clinical factors associated with the decision to perform surgical intervention are summarized in Table 3.

Ultrasound findings offered further insights: increased testicular size was seen in 28 out of 63 patients (44.4%; 48 missing data points), testicular heterogeneity was noted in 31 out of 65 patients (47.7%; 46 missing data points), absent vascular signals were detected in 31 out of 67 patients (46.3%; 44 missing data points), and the whirlpool sign of the spermatic cord was identified in 24 out of 61 patients (39.3%; 50 missing data points). All these ultrasound findings showed a positive correlation with testicular torsion, with the following p-values: increased testicular size (*p* = 0.037), testicular heterogeneity (*p* < 0.001), absent vascular signals (*p* < 0.001), and the whirlpool sign (*p* < 0.001) (Table 4).

In patients who did not undergo surgical intervention, both clinical and ultrasound follow-up were performed. None of these patients developed testicular hypotrophy or atrophy during the observation period. No patients required parenteral hormone replacement therapy during follow-up.

## 4. Discussion

Acute scrotal pain in children and adolescents requires prompt evaluation, as it may indicate testicular torsion, a surgical emergency in which the spermatic cord twists, compromising blood flow to the testis [10]. Without timely intervention, testicular torsion can result in ischemia, necrosis, infertility, and cosmetic deformities. The condition exhibits a bimodal age distribution, with incidence peaks during infancy and adolescence, and an estimated annual occurrence of approximately 3.8 cases per 100,000 children [11,12].

Common presenting symptoms include intense scrotal pain, nausea, and vomiting. However, these manifestations may overlap with other conditions such as torsion of a testicular appendage or epididymo-orchitis. As such, the evaluation of acute scrotum necessitates careful consideration of multiple differential diagnoses. Among these, torsion of the spermatic cord is the most critical, given its potential to compromise gonadal viability. Nonetheless, torsion of the testicular appendage and epididymo-orchitis must also be thoroughly assessed, underscoring the importance of precise clinical differentiation to ensure appropriate and timely management [13,14].

To enhance diagnostic accuracy in cases of acute scrotum, the TWIST score was developed as a clinical tool to support timely and informed decision-making [5]. This clinical scoring system allocates points based on specific findings: testicular swelling (2 points), firm testis (2 points), nausea or vomiting (1 point), an absent cremasteric reflex (1 point), and a high-riding testis (1 point). High TWIST scores (5–7 points) can directly indicate the need for surgery, thus reducing diagnostic delays and healthcare costs.

In this study, we examined key clinical indicators in patients presenting with acute scrotal pain, analyzing their correlation with symptom onset, patient age, and final diagnosis. A high riding testis and absence of the cremasteric reflex were strongly associated with testicular torsion, exhibiting high correlation coefficients with statistical significance (*p* < 0.0001).

These findings are consistent with those of Frohlich et al. [15], who emphasized the diagnostic value of an absent cremasteric reflex. While the TWIST score attributes only a single point to this sign, our logistic regression analysis identified it as a significantly stronger predictor of testicular torsion, with an Exp(B) value of 25.875. This suggests that an absent cremasteric reflex increases the likelihood of torsion of the spermatic cord by approximately 26-fold compared to its presence.

Furthermore, firm testis emerged as another crucial indicator, strongly linked to prolonged ischemia (*p* = 0.001). Moreover, a significant inverse correlation between testicular firmness and non-surgical management (*p* < 0.001) underscored the urgency of surgical operation in such cases. Scrotal swelling and hyperemia were commonly observed in patients with spermatic testicular torsion, likely reflecting the inflammatory response to ischemia. However, given the high prevalence of swelling in other conditions, such as orchi-epididymitis, its presence alone lacked diagnostic specificity, despite being noted in 92.2% of torsion cases.

Timely presentation is of paramount importance: patients presenting more than eight hours after symptom onset were significantly more likely to require orchiectomy, whereas earlier presentation was associated with higher rates of successful detorsion. Although the duration of pain at presentation was not statistically associated with a final diagnosis of spermatic cord torsion (*p* = 0.063), delayed presentation showed a significant correlation with surgical conditions (*p* = 0.049), possibly due to its more insidious pain profile, which may contribute to delayed medical attention.

In clinical scenarios where findings were ambiguous, further diagnostic evaluation proved essential. Doppler ultrasonography remains the gold standard, with hallmark features suggestive of spermatic cord torsion including absent or diminished testicular blood flow, “whirlpool sign,” heterogeneous echotexture, and testicular enlargement [15]. Nonetheless, reported delays in obtaining ultrasound examinations highlight the imperative for expedited imaging [13,16,17,18]. In cases where diagnostic uncertainty persists, ultrasonographic evaluation provides critical adjunctive information. Notably, increased testicular volume (*p* = 0.037) and parenchymal heterogeneity (*p* < 0.001) have emerged as strong sonographic predictors of spermatic cord torsion [4,19]. Among these, the absence of intratesticular blood flow is particularly significant, demonstrating a strong association with the need for surgical intervention (*p* < 0.001). This finding serves as a pivotal marker of testicular torsion or other emergent scrotal pathologies requiring immediate surgical management to prevent irreversible ischemic damage [4,6]. Conversely, the absence of these sonographic abnormalities tends to correlate with non-operative management strategies (*p* = 0.007). Additionally, the identification of the “whirlpool sign”, indicative of twisted spermatic cord structures, is highly predictive of testicular torsion and reinforces the need for prompt operative exploration (*p* < 0.001).

**Figure 1:**
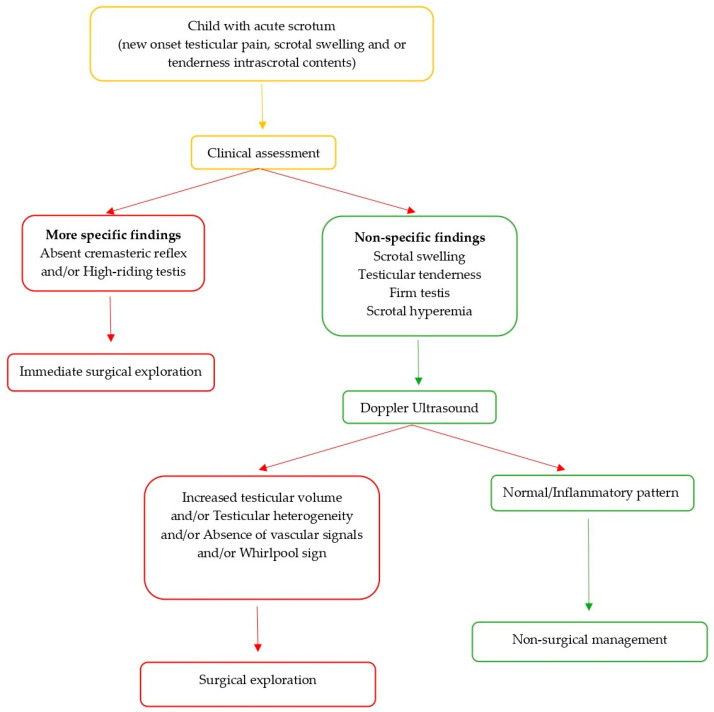
Schematic algorithm in pediatric acute scrotum.

On the basis of our results, we developed an algorithm that summarized the pathway that could be followed in acute scrotum management (Figure 1).

Educational interventions directed at both adolescents and healthcare professionals are essential to improving outcomes in acute scrotal emergencies. Increased awareness of the clinical hallmarks of torsion, along with the critical importance of timely medical assessment, can substantially reduce delays in diagnosis and enhance testicular salvage rates. Furthermore, improving emergency clinicians’ proficiency in recognizing key clinical and ultrasonographic indicators may lead to greater diagnostic accuracy, expedited surgical decision-making, and a reduction in morbidity associated with delayed intervention.

## 5. Conclusions

This study highlights the critical importance of early and accurate clinical assessment in patients presenting with acute scrotal pain, particularly in differentiating testicular torsion from other etiologies. Key clinical signs, such as the absence of the cremasteric reflex and a high-riding testis, were strongly associated with the need for surgical intervention, whereas their absence was more often linked to non-operative management. Timely presentation proved essential for testicular salvage, as delayed evaluation significantly increased the risk of orchiectomy. Additionally, ultrasonographic findings, including absent intratesticular blood flow and testicular heterogeneity, played a decisive role in guiding management when the clinical picture was inconclusive. Early recognition and prompt intervention remain paramount to improving outcomes and preventing irreversible testicular damage.

## Figures and Tables

**Table 1:** Frequency of clinical signs.

Clinical Sign	Patients (n)	Percentage (%)
Scrotal swelling	95/103	92.2
Tenderness on palpation	100/107	93.5
Firm testis	43/65	66.2
Scrotal hyperemia	65/77	84.4
Absence of the cremasteric reflex	25/38	65.8
High riding testis	13/41	31.7

**Table 2:** Percentage incidence related to spermatic cord torsion along with corresponding p-values.

	Spermatic Cord Torsion	
	Percentage	*p*-Value
Absence of the cremasteric reflex	65.8%	<0.0001
High riding testis	31.7%	<0.0001
Time of pain onset > 8 H	60.4%	0.200
Scrotal swelling	92.2%	0.890
Testicular tenderness	93.5%	0.549
Firm testis	66.2%	0.105
Scrotal hyperemia	84.4%	0.267

**Table 3:** Clinical factors associated with surgical treatment of acutum scrotum. Percentages reflect the presence of each sign or symptom.

	Surgical Treatment	
	Percentage	*p*-Value
Absence of the cremasteric reflex	84.6%	<0.01
High riding testis	58.8%	<0.01
Time of pain onset > 8 H	50.9%	0.026
Scrotal swelling	93%	0.752
Testicular tenderness	93.2%	0.912
Firm testis	73.7%	0.128
Scrotal hyperemia	78.4%	0.160

**Table 4:** Correlation between ultrasound findings and spermatic cord torsion.

Ultrasound Finding	Spermatic Cord Torsion
Increased testicular volume	*p* = 0.037
Testicular heterogeneity	*p* < 0.001
Absence of vascular signals	*p* < 0.001
Whirlpool sign	*p* < 0.001

## Data Availability

The data presented in this study are available upon request from the corresponding author. The data are not publicly available due to privacy and ethical restrictions.
